# Supplementary material for: Interrelated adolescent-level food insecurity and common mental health disorders in Harari Region, Ethiopia: A cross-sectional study
Source: PLoS One. 2026 May 7;21(5):e0348774. doi: 10.1371/journal.pone.0348774 (PMC13152165; doi:10.1371/journal.pone.0348774)
Supplement: Attachment — Submitted filename: Response to Reviewers.docx [file pone.0348774.s004.docx]

**Date:** 09/03/2026
**Manuscript ID:** PONE-D-25-41851

**Title:** Interrelated Adolescents’ food insecurity and common mental health disorders in Harari Region, Ethiopia: a cross-sectional study

**To: The Editor, PLOS ONE**

Thank you for the opportunity to revise our manuscript. We appreciate the constructive feedback provided by the editorial team and the reviewer. We have addressed each point step by step below and updated the manuscript accordingly.

**Part 1: Editor’s Comments**

1. Please ensure that your manuscript meets PLOS ONE's style requirements, including those for file naming.

**Response:** We have updated the manuscript to journal format, including the title page, affiliations, and headings, to strictly adhere to the PLOS ONE style templates. File names have been updated accordingly.

2. Please include captions for your Supporting Information files at the end of your manuscript, and update any in-text citations to match accordingly. Please see our Supporting Information guidelines for more information:

<http://journals.plos.org/plosone/s/supporting-information>.

**Response:** Captions for all Supporting Information files (e.g., S1 Data) have been added to the end of the manuscript. In-text citations have been cross-checked for consistency.

3. If the reviewer comments include a recommendation to cite specific previously published works, please review and evaluate these publications to determine whether they are relevant and should be cited. There is no requirement to cite these works unless the editor has indicated otherwise.

**Response:** We have reviewed the literature suggested by the reviewer (regarding updated SDG data and validated tools) and incorporated relevant citations as requested.

**Additional Editor Comments:**

After careful consideration and based on my evaluation and reviewer feedback, the topic is relevant, the sample size is large (n = 3,227 analysed), and the use of SEM adds analytical value. However, there are substantial methodological and reporting weaknesses that must be addressed before the manuscript can be considered publishable.

Please take my comments under consideration as follows:

1. Please clarify whether the construct is adolescent-level food insecurity or household food insecurity, and align title, aims, tools, and discussion accordingly.

**Response:** We have clarified that the study focuses on adolescent-level food insecurity. As our tool measures adolescent-level food insecurity or child-report or individual-level food insecurity, we assessed specific characteristic of individual as follow:  We asked adolescents directly at school about their own "food habit" and experiences, rather than asking a parent or head of household about the family's overall situation. We used questions that specifically describe about what the adolescents themselves experience (e.g., " Whether adolescents never experience (1), experience for a week (2), three weeks (3), or more than 21 days (4) is evaluated by the questions."), which is the hallmark of individual-level scales. Therefore, the title, objectives, tools section, and discussion have been aligned to reflect this distinction consistently.

2. In the method section, the design is cross-sectional, but no temporal sequencing is established, or mediation language implies causality. This is a serious limitation. Please remove causal/mediation language and reframe as associations consistent with potential mediation pathways. And please add stronger limitation section.

**Response:** We have removed all terms implying causality (e.g., "effect," "impact," "leads to") throughout the manuscript and replaced them with "association" or "pathway." We have reframed the mediation analysis now as a "potential association pathway." We have also expanded the limitations section to explicitly state that the cross-sectional nature of the data precludes any definitive causal inferences.

3. In Table 1 and CFA results several RMSEA values are poor including food insecurity RMSEA = 0.09 and KIDSCREEN RMSEA = 0.10 as well as CFI/TLI borderline in some models. Beyond that Cronbach alpha for dietary diversity = 0.62 (low). These raise concerns about construct validity. Please justify model fit thresholds, provide standardized factor loadings, and report composite reliability and AVE.

**Response:** We thank the reviewer for this important observation. While the AVE values for these constructs are below the traditional 0.50 threshold, we have retained them in the model based on established psychometric criteria and the specific nature of the variables under study. Our justification is three-fold:

According to Fornell and Larcker (1981), if the Average Variance Extracted (AVE) is less than 0.50 but the Composite Reliability (CR) is higher than 0.60, the convergent validity of the construct may still be considered adequate. In our study, all constructs meet this threshold:

- KIDSCREEN-10: CR = 0.82 (AVE = 0.36)
- Self-Esteem: CR = 0.76 (AVE = 0.36)
- SDQ-20: CR = 0.74 (AVE = 0.21)
- Dietary Diversity: CR = 0.62 (AVE = 0.22)

The structural integrity of our model is supported by strong global fit indices across all constructs. Specifically, the RMSEA values (ranging from 0.04 to 0.05 for most constructs) and SRMR (ranging from 0.02 to 0.05) indicate that the models are well-specified and fit the data accurately. High CFI and TLI values further confirm that the overall structure is robust, despite the high unique variance in individual items.

These are "heterogeneous" or "index-like" constructs. For example, in Dietary Diversity, the consumption of one food group does not theoretically necessitate the consumption of another. Similarly, the SDQ-25 aggregates distinct behavioural domains (emotional, conduct, hyperactivity, peer problem). In such cases, items are not expected to be highly redundant, which naturally results in a lower AVE while maintaining high overall reliability (CR).

All scales used are globally validated, standardized instruments. Deleting items to artificially inflate the AVE would compromise the content validity and the ability to compare our findings with existing literature. Given that the CR values are acceptable, the global fit is excellent, and the items are theoretically sound, the constructs demonstrate sufficient reliability and validity for inclusion in the final structural model.

4. There are numerous issues regarding grammar errors throughout, inconsistent terminology (common mental disorders / mental health disorders / illnesses), typographical errors (e.g., Adolfi → QoL, slfestm). Editing is must.

**Response:** In response to the reviewer’s feedback, we have conducted a thorough language edition of the entire manuscript. Specifically: We have corrected grammatical inconsistencies, improved sentence structure, and standardized technical terms (e.g., changing "pupils" to "students," "domicile" to "locality," and "chat" to "khat"). Both the Abstract and Conclusion sections were rewritten to ensure that our findings are presented as statistical correlates and significant risk markers rather than confirmed causal mechanisms. Terminology has been standardized to "Common Mental Disorders (CMD)." Typos like "Adolfi" (Quality of Life) and "slfestm" (self-esteem) have been corrected.

5. Please need Table formatting.

We have systematically revised all tables in the manuscript, in response to the request for improved table formatting and the inclusion of additional validity metrics. Specifically:

- Psychometric and Fit Indices: We have updated the tables to include Standardized Factor Loadings, Composite Reliability (CR), and Average Variance Extracted (AVE) for each construct to provide a comprehensive view of construct validity.
- Standardization of Numerical Reporting: All statistical values including factor loadings, Cronbach’s alpha, and fit indices (CFI, TLI, RMSEA, SRMR) have been standardized to two decimal places for consistency. P-values are reported to three decimal places.
- Alignment with Journal Standards: Following standard academic formatting (APA/Vancouver), we have removed all vertical lines and unnecessary shading. Tables now utilize the three-line rule: a top border, a header border, and a bottom border.
- Associational Terminology: Table titles and headers have been revised to use strictly associational language (e.g., 'Associations' instead of 'Effects') to accurately reflect the cross-sectional nature of the study design.
- Self-Explanatory Design: We have included detailed footnotes for all abbreviations (e.g., CMD, RMSEA, CFI) to ensure each table remains independently comprehensible to the reader."

**Part 2: Reviewer #1 Comments**

Is the manuscript presented in an intelligible fashion and written in standard English?
PLOS ONE does not copyedit accepted manuscripts, so the language in submitted articles must be clear, correct, and unambiguous. Any typographical or grammatical errors should be corrected at revision, so please note any specific errors here.

Reviewer #1: No

**Response:** We sincerely thank the reviewer for highlighting the need for improved linguistic clarity. We recognize that as an observational study, our initial draft contained several instances of ambiguous or causal phrasing that did not meet the rigorous standards of *PLOS ONE*. In response, the following actions were taken:

- Comprehensive Language Edition: The entire manuscript has undergone a professional-level language review to ensure it is written in standard, intelligible English. We have corrected all typographical errors, improved sentence structures, and standardized technical terminology (e.g., consistently using 'adolescents' instead of 'teenagers' and 'students' instead of 'pupils').
- Removal of Causal Ambiguity: We have systematically audited the manuscript to remove 'causal' language (e.g., 'impact of,' 'results in,' 'causes,' or 'leads to'). These have been replaced with strictly associational terms (e.g., 'associated with,' 'linked to,' 'correlated with,' or 'identified alongside') to accurately reflect the cross-sectional nature of the study.
- Structural Refinement: The Abstract, Results, and Conclusion sections were rewritten to ensure that findings are presented clearly and unambiguously as statistical correlates.
- Terminology Standardization: Cultural and regional terms have been standardized for an international audience (e.g., 'Chat' has been corrected to 'Khat' and 'domicile' to 'locality').
- Table and Data Presentation: All tables were reformatted for better intelligibility, including standardized decimal places and clear, self-explanatory headers.

Reviewer #1: I have some recommendations for the authors:

1. Please check the spelling, grammar, and sentence structures throughout the manuscript. Several sentences are excessively long and contain too much information, making them difficult to follow (e.g., lines 56-58; 72-76; 196-197).

**Response:** We have broken down long sentences throughout the manuscript to improve readability and flow.

2. Methods section in the Abstract: The study tool is unclearly described. For instance, it is not specified how many components it has, or what those components are. Additionally, it is unclear whether all or only some parts of the questionnaire have been validated.

**Response:** We have revised the Methods section of the Abstract to clearly specify the instruments used, including their item counts and validation status. We have explicitly noted that the study utilized internally and externally validated scales, including the HFIAS, KIDSCREEN-10, Rosenberg Self-Esteem Scale, and SDQ-25. For detail, we have added Table 1 in the Methods section (page 9) to provide a detailed summary of the specific tools used, their validation status, and the Cronbach's alpha values for each scale.

3. Introduction: References should be refined. The authors should prioritize the newest references for supporting information. For instance, the reference cited for information on Sustainable Development Goals (SDGs) in lines 55-56 is from 2016, which might be outdated.

**Response:** We sincerely thank the reviewer for the suggestion to prioritize more recent literature. We have updated the manuscript with the most recent global and regional statistics. We have systematically edited our reference list and added the most current available data. Specifically, we now cite the 2024/2025 State of Food Security (SOFI) report and the October 2024 WHO/UNICEF joint guidance on adolescent mental health. Additionally, the prevalence of childhood mental illness in Ethiopia has been refined using a 2023 meta-analysis, providing a more accurate reflection of the current regional burden (24.68%). We have prioritized research from the last five years (2020–2025) to support our discussion on the behavioural and psychosocial correlates of food insecurity among adolescents.

4. The reference list should be checked and corrected (e.g., the 3rd reference).

**Response:** The reference list has been edited. The 3rd reference and others have been formatted correctly according to the style required by the journal.

5. The data collection tool needs to be described more clearly. This includes providing a detailed list of specific tools used to measure each component (e.g., FIES, mental health disorders), along with information on their validation (both internationally and nationally). Similarly, the "Estimate of scale reliability" should be presented clearly, such as a summary of Cronbach's alpha for each scale used.

**Response:** We thank the reviewer for this constructive suggestion. We have thoroughly revised the 'Methods' section (see pages [7-9]) to provide a clearer description of the data collection instruments. Specifically, we have: Detailed each tool used (e.g., AFIAS, RSES, SDQ-25 and GSHS modules), including the specific number of items. Included a summary of the scale reliability (Cronbach's alpha) calculated from our study’s dataset to demonstrate internal consistency for each component." As requested, we have added Table 1 in the Methods section (page 9) to provide a detailed summary of the specific tools used, their validation status, and the Cronbach's alpha values for each scale.

6. A summary of the results for key variables such as FIES, quality of life, and mental health disorders should be presented. This would provide readers with a general overview of these issues relevant to the study objectives. For example, the authors used terms like "common mental health disorders" or "prevalent mental health disorders," but what data specifically supported these statements? What data showed that mental disorders are indeed prevalent or common in the study population?

**Response:** We appreciate the reviewer’s feedback. We have standardized the terminology to 'common mental disorders (CMD)' throughout the manuscript to reflect the specific category of disorders studied (e.g., anxiety and depression), rather than to imply their baseline prevalence. To provide further clarity on the actual status within our study population, we have added Table 2, which summarizes the variables and the prevalence of CMD observed in our findings (i.e.., the distribution, mean scores, and prevalence rates for the primary study outcomes, including food insecurity, mental health, and quality of life).

We believe these revisions significantly strengthen the manuscript and address all concerns. We look forward to your decision.

Sincerely,

Kasiye Shiferaw/Corresponding Author
