# Supplementary material for: Interrelated adolescent-level food insecurity and common mental health disorders in Harari Region, Ethiopia: A cross-sectional study
Source: PLoS One. 2026 May 7;21(5):e0348774. doi: 10.1371/journal.pone.0348774 (PMC13152165; doi:10.1371/journal.pone.0348774)
Supplement: Attachment — Submitted filename: Comments 03.4.2026.docx [file pone.0348774.s003.docx]

***Comments adolescent FI in Ethiopia***

**Abstract**

Define **"Common Mental Disorders (CMDs)"** at its first occurrence to ensure clarity for all readers.

Briefly specify the **sampling technique** (e.g., stratified or cluster sampling) to clarify the representativeness of the 3,326 participants

Report the **Model Fit Indices** (e.g., RMSEA, CFI, TLI) to validate the structural integrity of the SEM model before interpreting the path coefficients.

Include the **prevalence rates** of food insecurity and CMDs to provide a clear descriptive context alongside the regression results.

The use of the term 'co-occur' in the conclusion of the Abstract does not accurately reflect the nature of the SEM model; the author should replace it with phrases that indicate functional associations or predictive power (such as 'significantly associated with' or 'predict') to better align with the observed beta coefficients.

Line 142-147: Specify which primary outcome or exposure variable the **18.5% prevalence estimate** refers to (e.g., CMDs or food insecurity) to justify the sample size calculation.

"Remove the colon after **'specifically'** as it is grammatically redundant and disrupts the sentence flow."

Line 179-180; 187-188: "Remove **'it'** and rephrase for better flow, for example: 'A validated tool was used to measure **this condition**...' or simply '...to measure **adolescent-level food insecurity**. → Adolescent-level food insecurity was assessed using a validated scale covering the previous three months.

Line 189: The abbreviation **'HRQOL'** should be written in full as **'Health-Related Quality of Life'** at its first occurrence.

Please revise the sentence for grammatical completeness “Using a 4-point Likert scale from "strongly disagree" (1) to "strongly agree" (4), Rosenberg Self-Esteem Scale employed”

**Variables and measurements**

The description of the instruments remains fragmented. Specifically, for the **HFIAS adaptation**, please list the 5 items used and clarify if this abbreviated version has been formally validated in the Ethiopian context.

While the Cronbach's alpha values are provided, the authors should include a brief interpretation or reference for these values (e.g., indicating whether they represent 'acceptable' or 'good' internal consistency). This would provide readers with a clearer benchmark for the reliability of the scales used in the Ethiopian context.

The description of sociodemographic variables, particularly **'financial difficulties,'** is too general. Please specify how this was measured and categorized in your analysis? Additionally, clarify the categories used for **'place of residence'**.

The reliability of measuring 'financial difficulties' through adolescent self-reports is questionable, as students often lack a complete understanding of their household's economic status. I suggest the authors discuss this as a potential limitation or explain if more objective proxies (e.g., parental occupation or household assets) were used instead.

The **Ethical Considerations** section is somewhat repetitive, particularly the repeated use of 'written, informed, and signed consent.

The authors used **0.4** as the cut-off for factor loadings. While this is a common practice, please provide a supporting reference.

The **Data Processing and Analysis** section is currently too wordy and contains many textbook-style definitions that could be summarized. I suggest consolidating the descriptions of the four SEM models and streamlining the justification for the fit indices (specifically RMSEA and Cronbach’s alpha). Focus on the **actions taken** and the **thresholds applied** rather than explaining the underlying statistical theories. This will make the methodology much more concise and easier for the reader to follow.

**Results**

Table 2 is missing the percentage symbol (%) in the second column for rows 3 to 5 (Mental Health, Quality of Life, and Self-Esteem).

The implied total sample size (N) varies slightly between variables (e.g., 3225 vs 3228). Please clarify the total N and account for any missing data in a table footnote.

The 'Interpretation/Status' column repeats the numerical data.

The classification of 'moderate to severe food insecurity' (14.51%) in Table 2 is not clearly linked to the 5-item HFIAS scale described in the Methods. Please specify the cut-off scores or the scoring algorithm used to categorize participants into these specific levels, especially since a non-standard 5-item version was employed.

Table 3 is missing the footnote for 'CR' (Composite Reliability). Please provide a clear definition and the threshold used for this indicator below the table.

**Discussion**

The Discussion should go beyond comparing prevalence rates with previous studies. Specifically, I suggest the authors elaborate on why **Self-Esteem** emerged as the strongest predictor (beta = -0.41) in the SEM model. Discussing the psychological mechanism through which food insecurity erodes an adolescent's self-worth is more valuable than repetitive literature comparisons.

The current recommendations are somewhat generic. Given that the study focuses on school-going adolescents, the authors should propose specific interventions such as **targeted school feeding programs** or **school-based psychological support** to bolster self-esteem and quality of life, rather than broad macroeconomic policies.

The authors should clearly discuss the reduction of the AFI coefficient from Model I to Model IV. This 'attenuation' is a key finding that demonstrates how psychosocial factors partially mediate the link between hunger and mental health."
