# Supplementary material for: Interrelated adolescent-level food insecurity and common mental health disorders in Harari Region, Ethiopia: A cross-sectional study
Source: PLoS One. 2026 May 7;21(5):e0348774. doi: 10.1371/journal.pone.0348774 (PMC13152165; doi:10.1371/journal.pone.0348774)
Supplement: Attachment — Submitted filename: Response_to_Reviewers_auresp_2.docx [file pone.0348774.s005.docx]

**Dear Editor and Reviewers,**

Thank you for providing us with the opportunity to revise our manuscript entitled **"**Interrelated adolescent-level food insecurity and common mental health disorders in Harari Region, Ethiopia: A cross-sectional study**"** (Manuscript ID: PONE-D-25-41851R1). We would like to express our sincere appreciation to the reviewers for their thorough and constructive feedback.

We have carefully considered all the comments and have revised the manuscript accordingly. We believe that these revisions have significantly enhanced the clarity, depth, and overall quality of our work.

Detailed point-by-point responses to the reviewers' comments are provided below, along with descriptions of the corresponding revisions. For your convenience, all changes have been marked in the manuscript using the 'Track Changes' feature.

**Part 1: Response to Journal Requirements:**

**Reviewer Concern:** If the reviewer comments include a recommendation to cite specific previously published works, please review and evaluate these publications to determine whether they are relevant and should be cited. There is no requirement to cite these works unless the editor has indicated otherwise.

**Action Taken:** We appreciate the reviewer’s suggestion to include these references. After reviewing the recommended publications, we agree they provide valuable context to variable and measurement. We have added citations for **Jebena et al. (2017) and Belachew et al. (2013)** on page 7 and updated the bibliography accordingly.

**Reviewer Concern:** Please review your reference list to ensure that it is complete and correct. If you have cited papers that have been retracted, please include the rationale for doing so in the manuscript text, or remove these references and replace them with relevant current references. Any changes to the reference list should be mentioned in the rebuttal letter that accompanies your revised manuscript. If you need to cite a retracted article, indicate the article’s retracted status in the References list and include

**Action Taken:** We conducted a thorough audit of our 79 references. **No retracted papers are cited.** We have updated the DOIs for Ref #12 and Ref #75 and ensured Ref #34 reflects the most current metadata. Furthermore, we integrated new high-authority citations to provide a robust theoretical basis for our statistical thresholds.

**Reviewer Concern:** 5. Is the manuscript presented in an intelligible fashion and written in standard English? PLOS ONE does not copyedit accepted manuscripts, so the language in submitted articles must be clear, correct, and unambiguous. Any typographical or grammatical errors should be corrected at revision, so please note any specific errors here.

**Action Taken:** The entire manuscript has undergone a comprehensive professional proofreading. We have corrected all specific errors noted (e.g., redundant colons, incomplete sentences regarding the Rosenberg Scale). We have restructured fragmented sections in the "Methods" and "Results" to ensure a cohesive narrative flow that meets *PLOS ONE*’s standards for clear and unambiguous academic English.

**Part 2: Point-by-Point Response to Reviewer #1**

- **Abstract:**

**Reviewer Concern:** Define "Common Mental Disorders (CMDs)" at its first occurrence to ensure clarity for all readers.

**Action Taken:** We have now defined "Common Mental Disorders (CMDs)" at first occurrence to ensure the scope of our outcome variable (anxiety and depression) is clear to all readers.

**Reviewer Concern:** Briefly specify the sampling technique (e.g., stratified or cluster sampling) to clarify the representativeness of the 3,326 participants

**Action Taken:** We clarified the **multistage sampling** strategy to demonstrate the representativeness of the 3,326 participants, ensuring the findings can be generalized to the school-going population in the region.

**Reviewer Concern:** Report the Model Fit Indices (e.g., RMSEA, CFI, TLI) to validate the structural integrity of the SEM model before interpreting the path coefficients.
**Action Taken:** We added the **Model Fit Indices (RMSEA = 0.03, CFI = 0.90, TLI = 0.89, SRMR = 0.05)**. By including these, we demonstrate the mathematical validity of our SEM model before discussing the path coefficients.

**Reviewer Concern:** Include the prevalence rates of food insecurity and CMDs to provide a clear descriptive context alongside the regression results.

**Action Taken:** Prevalence for adolescents’ food insecurity (**14.50%**) and CMDs (**22.93%**) were added to provide a baseline for the regression results.

**Reviewer Concern:** The use of the term 'co-occur' in the conclusion of the Abstract does not accurately reflect the nature of the SEM model; the author should replace it with phrases that indicate functional associations or predictive power (such as 'significantly associated with' or 'predict') to better align with the observed beta coefficients.

**Action Taken:** Following the reviewer's excellent point, we removed the passive term "co-occur" and replaced it with functional/predictive language (e.g., "predicts," "significantly associated with") to align with the beta-coefficient results of the SEM.

- **Methods:**

**Reviewer Concern:** Line 142-147: Specify which primary outcome or exposure variable the 18.5% prevalence estimate refers to (e.g., CMDs or food insecurity) to justify the sample size calculation.
**Action Taken:** We specified that the **18.5% prevalence estimate** refers specifically to **food insecurity** (our primary exposure), providing the necessary justification for our power calculation.

**Reviewer Concern:** "Remove the colon after 'specifically' as it is grammatically redundant and disrupts the sentence flow."

**Action Taken:** we have removed colon after 'specifically'.

**Reviewer Concern:** Line 179-180; 187-188: "Remove 'it' and rephrase for better flow, for example: 'A validated tool was used to measure this condition...' or simply '...to measure adolescent-level food insecurity. → Adolescent-level food insecurity was assessed using a validated scale covering the previous three months.

**Action Taken:** We addressed the "fragmented" description by listing the **5 specific HFIAS items** used. Crucially, we cited **Jebena et al. (2017) and Belachew et al (2013) (Ref #32&42)** to prove that this abbreviated tool is formally validated and culturally appropriate for the Ethiopian context.

**Reviewer Concern:** Line 189: The abbreviation 'HRQOL' should be written in full as 'Health-Related Quality of Life' at its first occurrence.

**Action Taken:** We agree with the reviewer’s suggestion. We have now defined the abbreviation at its first mention in the text (Line 197).

**Reviewer Concern:** Please revise the sentence for grammatical completeness “Using a 4-point Likert scale from "strongly disagree" (1) to "strongly agree" (4), Rosenberg Self-Esteem Scale employed”
**Action Taken:** We have revised this sentence for clarity and grammatical correctness.

**Reviewer Concern:** While the Cronbach's alpha values are provided, the authors should include a brief interpretation or reference for these values (e.g., indicating whether they represent 'acceptable' or 'good' internal consistency). This would provide readers with a clearer benchmark for the reliability of the scales used in the Ethiopian context.

**Action Taken:** Thank you for this insightful suggestion. We agree that providing a clear benchmark for the internal consistency of the adapted AFI scale (the primary exposure variable) is essential for readers to evaluate its reliability within this specific context. Accordingly, we have updated the **Methods** section (under the **" Variables and measurements "** subtopic) on page 8 and 9, line 215-223 to include the appropriate interpretation. We also added qualitative interpretations for all alpha values (e.g., **0.82 as "good," 0.74 as "acceptable"**), providing a clear reliability benchmark for our measurements.

**Reviewer Concern:** Variables and measurements

The description of the instruments remains fragmented. Specifically, for the HFIAS adaptation, please list the 5 items used and clarify if this abbreviated version has been formally validated in the Ethiopian context.

**Action Taken:** We have revised the "Variables and Measurements" section to include the full list of the 5 HFIAS items used in this study. This abbreviated version was adapted to focus on the items most sensitive to the local adolescent context, as supported by previous validation work in Ethiopia.

**Action Taken:** Now explicitly defined as **Urban vs. Rural** using official **Ethiopian Central Statistical Agency (CSA)** population and administrative criteria (Ref #46).

**Reviewer Concern:** The description of sociodemographic variables, particularly 'financial difficulties,' is too general. Please specify how this was measured and categorized in your analysis? Additionally, clarify the categories used for 'place of residence'.
The reliability of measuring 'financial difficulties' through adolescent self-reports is questionable, as students often lack a complete understanding of their household's economic status. I suggest the authors discuss this as a potential limitation or explain if more objective proxies (e.g., parental occupation or household assets) were used instead.

**Action Taken:** We acknowledge the reviewer’s concern regarding the reliability of adolescent-reported financial status. While objective measures (e.g., parental income) are ideal, research indicates that adolescents’ subjective perception of financial stress is a unique and often stronger predictor of their mental health than objective parental reports supported by Davisson *et al*. (2025) (Ref #47). We have clarified the measurement process in the Methods section on page 10 and added this point to the Limitations section on page 21, noting that adolescents in our context provide a reliable proxy for household economic strain when direct parental data is unavailable. Clarified as a 3-level perceptual variable (Never, Sometimes, Always).

**Reviewer Concern:** The authors used 0.4 as the cut-off for factor loadings. While this is a common practice, please provide a supporting reference.

**Action Taken:** We justified the **0.4 cut-off** by citing **Hair et al. (2010)** (Ref #45), clarifying that this threshold ensures practical significance and accounts for at least 16% of the variance in each item.

**Reviewer Concern:** The Data Processing and Analysis section is currently too wordy and contains many textbook-style definitions that could be summarized. I suggest consolidating the descriptions of the four SEM models and streamlining the justification for the fit indices (specifically RMSEA and Cronbach’s alpha). Focus on the actions taken and the thresholds applied rather than explaining the underlying statistical theories. This will make the methodology much more concise and easier for the reader to follow.

**Action Taken:** We have streamlined the 'Data Processing and Analysis' section by removing theoretical definitions of Structural Equation Modeling (SEM) and fit indices. The revised text now focuses specifically on our four-model sequential approach and the applied statistical thresholds (e.g., RMSEA). This adjustment clarifies the methodology and improves the overall flow of the section, making the methodology much easier to follow.

**Reviewer Concern:** The Ethical Considerations section is somewhat repetitive, particularly the repeated use of 'written, informed, and signed consent.

**Action Taken:** We have revised the "Ethical Considerations" section to eliminate repetitive phrasing. The description of the consent process has been consolidated to clearly state that written informed consent was obtained from all participants (and their guardians, where applicable) without redundant terminology

- **Results:**

**Reviewer Concern:** Table 2 is missing the percentage symbol (%) in the second column for rows 3 to 5 (Mental Health, Quality of Life, and Self-Esteem).

**Action Taken:** We thank the reviewer for identifying this oversight. We have corrected Table 2 by adding the percentage symbols (%) to the specified rows (Mental Health, Quality of Life, and Self-Esteem) to ensure consistency and clarity.

**Reviewer Concern:** The implied total sample size (N) varies slightly between variables (e.g., 3225 vs 3228). Please clarify the total N and account for any missing data in a table footnote.

**Action Taken:** We thank the reviewer for this observation. We have verified the dataset and confirmed that the total number of participants included in the final analysis is 3,227. The slight variations noted may be due to minimal missing data (less than 0.1%) for specific variables. We have now standardized the reporting in Table 2 and added a footnote to clarify how missing data was handled.

**Reviewer Concern:** The 'Interpretation/Status' column repeats the numerical data.

**Action Taken:** We removed the redundant "Interpretation/Status" column. Status labels (e.g., "Abnormal range," "Low self-esteem") are now integrated into category names to simplify the data presentation.

**Reviewer Concern:** The classification of 'moderate to severe food insecurity' (14.51%) in Table 2 is not clearly linked to the 5-item HFIAS scale described in the Methods. Please specify the cut-off scores or the scoring algorithm used to categorize participants into these specific levels, especially since a non-standard 5-item version was employed.

**Action Taken:** We thank the reviewer for this important request for clarification. The 5-item HFIAS used in this study was adapted from the Jimma Longitudinal Family Survey of Youth (JLFSY) framework, as detailed by Jebena et al. (2017) and Belachew et al. (2013). This abbreviated version was specifically designed and validated for the Ethiopian adolescent context to capture the most culturally relevant dimensions of food access (worry, quality, and quantity) while reducing respondent burden. We have updated the Methods section to include the specific scoring algorithm and cut-off points used to define "moderate to severe" food insecurity.

**Reviewer Concern:** Table 3 is missing the footnote for 'CR' (Composite Reliability). Please provide a clear definition and the threshold used for this indicator below the table.

**Action Taken:** Added a footnote defining Composite Reliability (CR > 0.70) and Average Variance Extracted (AVE > 0.50), ensuring all SEM indicators are transparently reported.

- **Discussion:**

**Reviewer Concern:** The Discussion should go beyond comparing prevalence rates with previous studies. Specifically, I suggest the authors elaborate on why Self-Esteem emerged as the strongest predictor (beta = -0.41) in the SEM model. Discussing the psychological mechanism through which food insecurity erodes an adolescent's self-worth is more valuable than repetitive literature comparisons.

**Action Taken: The Mechanism of Self-Esteem** (beta = -0.41)**:** We expanded this section to discuss the **psychological "Internalized Stigma" mechanism**. We argue that food insecurity erodes self-worth through social comparison and shame in the school environment, which acts as the primary driver for CMDs.

**Reviewer Concern:** The current recommendations are somewhat generic. Given that the study focuses on school-going adolescents, the authors should propose specific interventions such as targeted school feeding programs or school-based psychological support to bolster self-esteem and quality of life, rather than broad macroeconomic policies.

**Action Taken:** We pivoted from broad macroeconomic suggestions to specific school-based interventions. We now propose targeted school feeding and on-site psychological counselling, which address both the physical and psychosocial mediators identified in our study (line 440-446; page 20).

**Reviewer Concern:** The authors should clearly discuss the reduction of the AFI coefficient from Model I to Model IV. This 'attenuation' is a key finding that demonstrates how psychosocial factors partially mediate the link between hunger and mental health.

**Action Taken:** We added a dedicated analysis of the reduction in the AFI coefficient from Model I to IV. We explain this "attenuation" as clear evidence of partial mediation, demonstrating that psychosocial factors are the "channels" through which hunger impacts the adolescent mind (line 434-439; page 20).

We believe these comprehensive revisions demonstrate our commitment to scientific excellence and fully address the reviewers' concerns. We hope the manuscript is now suitable for publication in *PLOS ONE*.

Sincerely,

Kasiye Shiferaw (PhD)

Corresponding Author
